# Supplementary material for: Animal abuse by falsification–Recognition amongst the veterinary profession in The Netherlands
Source: PLoS One. 2026 Apr 8;21(4):e0345067. doi: 10.1371/journal.pone.0345067 (PMC13061241; doi:10.1371/journal.pone.0345067)
Supplement: S1 File — (DOCX) [file pone.0345067.s001.docx]

**S1 File. Survey items on Animal Abuse by Falsification (AAF)**

Questionnaire

Are you familiar with one or both of below terms? (Yes, No, Don’t know)

-Animal abuse by fabricated or induced illness

-Munchausen syndrome by Proxy

We will hereafter define Animal Abuse by Fabricated or induced Illness (AAF) as:

An induced/fabricated condition in an animal which has been induced/fabricated by a caretaker, such as the owner. This caretaker has a sickly (morbid) need for attention by making an animal ill or pretending such illness.

Are you familiar with this phenomenon? (Yes, No, Don’t know)

Do you see AAF cases in your veterinary practice? (Yes, with certainty; Yes, likely, No, Don’t know)

If you see AAF cases in your veterinary practice, how often on a yearly base do you see:

- Cases of suspected AAF? Which animals are involved in the cases?

- Cases of confirmed AAF? Which animals are involved in the cases?

If you do not see AAF cases on a yearly base, have you ever in your career seen (an) AAF case(s)? (Yes, No, Don’t know)

- Suspected AAF

- Confirmed AAF (confirmed myself/ confirmed with a network partner)

If yes, how many and which animals were involved in the cases?

Which signs would make you think of AAF? (Open)

If you come/came across AAF, have you ever worked with a network partner, such as the police, to report the (suspected) case as animal abuse? (Yes, No, Don’t know)

If you come/came across AAF, have you ever reported AAF as animal abuse? (Yes, No, Don’t know)

What would help you to report AAF? (Open)

Which would be barriers to report AAF?

(Concerns about breaking client confidentiality; Concerns on litigation; Lack of knowledge of available resources; Lack of accepted standards in identification; A perception no action will be taken; Inexperience in dealing with misleading information provided by client; Concern that reporting may compromise safety of victim; Concern on physical retaliation by perpetrator; Concern that client will leave the clinic; Other, being [Open])

How likely would you rate each of below as a sign of AAF? (Highly unlikely, moderately unlikely, neutral, moderately likely, highly likely)

Signs and symptoms regarding animal and medical situation

Inexplicable medical symptoms

Unlikely medical history

Incompatibilities between medical history and clinical findings

Persistent or recurrent illnesses for which a cause cannot be found

Repeated hospitalisations and vigorous medical/veterinary evaluations of victim without definitive diagnoses

Recurrent illnesses in which poisoning may factor in

Recurrent illnesses in which suffocation may factor in

Recurrent illnesses in which nutrition, nutritional absorption, nutritional state may factor in

Gastro intestinal complaints for more than two weeks without a definitive diagnosis

Neurological complaints, e.g. epileptiform activity, lesser alertness (incl. coma)

Physiologic or laboratory parameters are noticeable or not fitting with patient profile

Erratic or toxic drug blood levels

Difficulty of diagnosis, rare or unsuspected disease pattern

Inexplicable intolerance of treatment or poor response to treatment

Recovery of animal/symptoms when hospitalized

Poor recovery if animal is with client

Relatively many animals deceased with client

Signs and symptoms regarding client behaviour

Much knowledge of the presented illness or generally of the medical/veterinary field

Resistance of client to (possibly effective) therapy suggestions

Little concern expressed over painful examinations/surgery

Relatively often visiting the clinic with this or multiple animals

Relatively often talking about illness in this or multiple animals

Relatively often talking about care burden or death of animal(s)

Relatively often talking about own illness or illness of relatives

Unexpected response in communication, such as anger upon referral

Sudden withdraw from treatment or staying away from clinic

Repeatedly presenting animal at various clinics

Expression of concern by relatives or other professionals

Perpetrator is known to have provided false information

How likely would you rate a combination of signs as indicative of AAF? (Highly unlikely, moderately unlikely, neutral, moderately likely, highly likely)

- Two signs

- Three to five signs

- More than five signs

A combination of specific signs, being (Open)
