# Supplementary material for: Animal abuse by falsification–Recognition amongst the veterinary profession in The Netherlands
Source: PLoS One. 2026 Apr 8;21(4):e0345067. doi: 10.1371/journal.pone.0345067 (PMC13061241; doi:10.1371/journal.pone.0345067)
Supplement: S2 Table — (DOCX) [file pone.0345067.s002.docx]

**S2 Table. Literature sources for Animal Abuse by Falsification (AAF) signs and symptoms**

| **Signs and symptoms regarding animal and medical situation** | **Oxley & Feldman, 2016** | **Munro & Thrusfield, 2001** | **Bass & Glaser, 2014** | **Geist et al., 2024** | **Jaghab et al., 2006** | **Shaw et al., 2008** |
| --- | --- | --- | --- | --- | --- | --- |
| Inexplicable medical symptoms | Unusual symptoms, signs or hospital/veterinary courses that do not make clinical sense. Victim’s appearance is inconsistent with illness | Clinical signs reported by the owner, but with no specific corroborating clinical evidence; postmortem examination refused | Biologically unlikely history of events |  | Dramatic or atypical presentation. Vague and inconsistent details, although possibly plausible on the surface. Admission circumstances that do not conform to an identifiable medical or mental disorder | One or more unexplained medical symptoms that do not respond well to treatment |
| Unlikely medical history | Discrepancies between history and clinical findings. Extreme rarity of the suspected disease or disease pattern, or a merely descriptive diagnosis. Experienced physicians/veterinarians state: ‘I’ve never seen a case like it before’ |  | Exaggeration, false reporting, misconstruing of real events on the basis of mistaken belief about their meaning | Inconsistencies in medical history | Fluctuating clinical course, including rapid development of complications or a new pathology if the initial workup findings prove negative. Long medical record with multiple admissions at various hospitals in different cities | Suspected medical problem is rare or unusual |
| Incompatibilities between medical history and clinical findings |  | Clinical signs reported and 'pretreated' by the owner, but with no clinical evidence on examination |  |  |  | Physical or laboratory findings that are discrepant or inconsistent with the history |
| Persistent or recurrent illnesses for which a cause cannot be found | Persistent or recurrent illnesses for which a cause cannot be found | A series of incidents, over a number of years |  | Unexplained symptoms |  |  |
| Repeated hospitalisations and vigorous medical/veterinary evaluations of victim without definitive diagnoses | Repeated hospitalisations and vigorous medical/veterinary evaluations of victim without definitive diagnoses | Repeated requests for treatment |  |  | Long medical record with multiple admissions at various hospitals in different cities |  |
| Recurrent illnesses in which poisoning may factor in |  | More common clinical presentations: bleeding (haematuria and haematemesis), seizures, CNS depression (drowsiness and coma), apnoea, failure to thrive, diarrhoea, vomiting, fever, rashes, hypertension. Conviction of the owner for attempted poisoning of his child (the owner’s dogs also had been poisoned by him previously) | Inducing of signs or illness in the child by, for example, poisoning or overmedication (e.g., laxatives, salt), suffocating, starving |  | Methods of induction of symptoms: Smothering or suffocating, Pushing fingers down the throat, Using laxatives or other drugs, Swallowing or injecting hazardous substances, Scratching or deliberating injuring the skin, Poking with a small or sharp instrument, Interfering with test samples |  |
| Recurrent illnesses in which suffocation may factor in |  | More common clinical presentations: bleeding (haematuria and haematemesis), seizures, CNS depression (drowsiness and coma), apnoea, failure to thrive, diarrhoea, vomiting, fever, rashes, hypertension | Inducing of signs or illness in the child by, for example, poisoning or overmedication (e.g., laxatives, salt), suffocating, starving |  | Methods of induction of symptoms: Smothering or suffocating, Pushing fingers down the throat, Using laxatives or other drugs, Swallowing or injecting hazardous substances, Scratching or deliberating injuring the skin, Poking with a small or sharp instrument, Interfering with test samples |  |
| Recurrent illnesses in which nutrition, nutritional absorption, nutritional state may factor in |  | More common clinical presentations: bleeding (haematuria and haematemesis), seizures, CNS depression (drowsiness and coma), apnoea, failure to thrive, diarrhoea, vomiting, fever, rashes, hypertension. | Inducing of signs or illness in the child by, for example, poisoning or overmedication (e.g., laxatives, salt), suffocating, starving |  | Methods of induction of symptoms: Smothering or suffocating, Pushing fingers down the throat, Using laxatives or other drugs, Swallowing or injecting hazardous substances, Scratching or deliberating injuring the skin, Poking with a small or sharp instrument, Interfering with test samples |  |
| Gastro intestinal complaints for more than two weeks without a definitive diagnosis | (Gastrointestinal (GI) cases) chronic diarrhoea with or without vomiting for more than two weeks. (GI cases) chronic vomiting and/or diarrhoea without definitive diagnosis | More common clinical presentations: bleeding (haematuria and haematemesis), seizures, CNS depression (drowsiness and coma), apnoea, failure to thrive, diarrhoea, vomiting, fever, rashes, hypertension |  |  |  |  |
| Neurological complaints, e.g. epileptiform activity, lesser alertness (incl. coma) |  | More common clinical presentations: bleeding (haematuria and haematemesis), seizures, CNS depression (drowsiness and coma), apnoea, failure to thrive, diarrhoea, vomiting, fever, rashes, hypertension. Clinical signs actually present: haematuria, restlessness, fits, fractures, abdominal bruising |  |  |  |  |
| Physiologic or laboratory parameters are noticeable or not fitting with patient profile | Physiologic or laboratory parameters are consistent with induced illness | Very abnormal electrolytes |  |  |  | Physical or laboratory findings that are discrepant or inconsistent with the history |
| Erratic or toxic drug blood levels | Erratic drug levels. Toxic drug levels on more than one occasion |  |  |  | Methods of induction of symptoms: Smothering or suffocating, Pushing fingers down the throat, Using laxatives or other drugs, Swallowing or injecting hazardous substances, Scratching or deliberating injuring the skin, Poking with a small or sharp instrument, Interfering with test samples |  |
| Difficulty of diagnosis, rare or unsuspected disease pattern | Extreme rarity of the suspected disease or disease pattern, or a merely descriptive diagnosis. The presumptive diagnosis is based solely on perpetrator report |  |  | Unexplained symptoms |  | Suspected medical problem is rare or unusual |
| Inexplicable intolerance of treatment or poor response to treatment | Persistent failure of a victim to tolerate or respond to medical/veterinary therapy without clear cause |  | An inexplicably poor response to prescribed drugs or other treatment | No improvement despite treatment. Unusual pattern of illness, especially when these illnesses coincide with a specific caregiver in attendance |  | One or more unexplained medical symptoms that do not respond well to treatment |
| Recovery of animal/symptoms when hospitalized | Illness abates when the victim is separated from the perpetrator (‘separation test’) | The symptoms and signs diminish or cease when the child is separated from the perpetrator. Recovery after hospitalisation (i.e., after separation from the owner) |  |  | Symptoms or behaviours only present when the patient is being observed | Symptoms resolve when the child is hospitalized or separated from the parents |
| Poor recovery if animal is with client | Episodes of illness begin when the perpetrator is or has recently been alone with the victim. Suspected interference with an orthopaedic pin |  | Reported symptoms and signs are only observed, or appear in the presence of, the parent or carer | No improvement despite treatment |  | Symptoms do not occur in absence of parent, or increase in presence of parent |
| Relatively many animals deceased with client | Unexplained illness or death in one or more siblings/pets | Serial deaths of pets, in unexplained and suspicious circumstances |  |  |  | Family history of similar sibling illnesses or unexplained sibling deaths |
|  |  |  |  |  |  |  |
| **Signs and symptoms regarding client behaviour** |  |  |  |  |  |  |
| Much knowledge of the presented illness or generally of the medical/veterinary field | Perpetrator has veterinary/medical or nursing training or access to illness models |  |  | Excessive medical knowledge. Overinvolvement with medical staff, showing unusual urgency, insistence, or understanding of medical procedures and treatments | Knowledge of textbook descriptions of illness. An unusual grasp of medical terminology. Employment in a medically related field | Parent who is very medically knowledgeable. Interest in medical details and enjoyment of hospital environment. Enjoyment of interacting with physicians. Interest in medical details of other patients. Parent who works in health care field or who expresses interest in doing so |
| Resistance of client to (possibly effective) therapy suggestions | Perpetrator refutes or is angered by negative test results or other negative findings, is noncompliant with treatment, or is disruptive in the clinic |  | Despite a definitive clinical opinion being reached, various opinions from both primary and secondary care are sought and disputed by the parent or carer, and the child continues to be presented for investigation and treatment with a range of signs and symptoms | Reluctance to have the dependent seen by alternative or independent healthcare professionals |  |  |
| Little concern expressed over painful examinations/surgery | A perpetrator who welcomes medical tests of the victim, even when painful |  |  |  | Acceptance, with equanimity, of the discomfort and risk of diagnostic procedures and risk of surgery | Apparent satisfaction when child is ill or hospitalized. Parent who is unusually calm in face of serious medical difficulties. Parent who demands further intervention, procedures, or second opinions |
| Relatively often visiting the clinic with this or multiple animals |  | Suspected attention-seeking behaviour by the owner. Repeated requests for treatment |  | Frequent medical visits |  |  |
| Relatively often talking about illness in this or multiple animals |  |  | New symptoms are repeatedly reported |  |  | Enjoyment of telling the medical history |
| Relatively often talking about care burden or death of animal(s) |  |  |  | Dramatization of symptoms |  |  |
| Relatively often talking about own illness or illness of relatives | Caregiver has features of Munchausen syndrome (multiple diagnoses, surgeries, and/or hospitalisations with no compelling diagnosis). Frequent comparisons of the victim’s medical problems to those of the perpetrator | Suspected attention-seeking behaviour by the owner |  |  |  | Parent who reports his or her own past history of similar or unexplained medical symptoms. Parent who reports multiple dramatic or unrealistic life events |
| Unexpected response in communication, such as anger upon referral |  | The perpetrator, at least initially, denies inventing or causing symptoms or signs |  | Defensiveness when questioned about the condition. Reluctance to have the dependent seen by alternative or independent healthcare professionals | Reluctance by the patient to allow healthcare professionals to meet with or talk to family members, friends, and prior healthcare providers. Controlling, hostile, angry, disruptive, or attention-seeking behaviour during hospitalization |  |
| Sudden withdraw from treatment or staying away from clinic | Caregiver demands that victim leaves hospital against medical advice or insists on transfer |  |  |  |  |  |
| Repeatedly presenting animal at various clinics | Care at more than one veterinary clinic/hospital within six months. There are consultations with one or more subspecialists. Victim has been to numerous health care/veterinary professionals without a resolution or even sustained improvement | Frequent change of address and veterinary practice |  |  | Long medical record with multiple admissions at various hospitals in different cities | Parent who transfers care between multiple institutions to pursue further diagnostic workup or treatment (“doctor shopping”) |
| Expression of concern by relatives or other professionals |  |  | Expression of concern by relatives or other professionals |  |  |  |
| Perpetrator is known to have provided false information | Perpetrator is known to have provided false information |  |  | History of false or extraordinary claims about their health or that of family members | Pseudologia fantastica |  |
