# Supplementary material for: Animal abuse by falsification–Recognition amongst the veterinary profession in The Netherlands
Source: PLoS One. 2026 Apr 8;21(4):e0345067. doi: 10.1371/journal.pone.0345067 (PMC13061241; doi:10.1371/journal.pone.0345067)
Supplement: S3 Table — (DOCX) [file pone.0345067.s003.docx]

**S3 Table. Participant details**

| **Gender** | **% (N)** |
| --- | --- |
| Female | 90.91% (N=80) |
| Male | 9.09% (N=8) |
| Non-binary | 0 |
| Other | 0 |
| Prefer not to say | 0 |
| **Age** |  |
| 18-35 years | 35.23% (N=31) |
| 35-55 years | 51.14% (N=45) |
| >55 years | 13.64% (N=12) |
| Prefer not to say | 0 |
| **Years in veterinary practice** |  |
| 0-5 years | 23.86% (N=21) |
| 5-15 years | 29.55% (N=26) |
| 15-25 years | 29.55% (N=26) |
| >25 years | 17.05% (N=15) |
| **Practice type** |  |
| Companion animals | 88.64% (N=78) |
| Horses | 3.41% (N=3) |
| Production animals | 0 |
| Mixed | 7.95% (N=7) |
| **Profession** |  |
| Para veterinary | 68.18% (N=60) |
| Veterinary | 31.28% (N=28) |
| **Have you ever received training or education on AAF?** |  |
| Yes | 11.36% (N=10) |
| No | 88.64% (N=78) |
