# Supplementary material for: Animal abuse by falsification–Recognition amongst the veterinary profession in The Netherlands
Source: PLoS One. 2026 Apr 8;21(4):e0345067. doi: 10.1371/journal.pone.0345067 (PMC13061241; doi:10.1371/journal.pone.0345067)
Supplement: S4 Table — (DOCX) [file pone.0345067.s004.docx]

**S4 Table. Animal Abuse by Falsification (AAF) signs and symptoms mentioned by participants to the open question on recognition: ‘Which signs would make you think of AAF?’**

The C’s (for ‘code’) with a number in the top row correspond with the codes assigned and listed in the table at the bottom of this file. When a ‘1’ is visible in a field, this indicates that the code was attributed based on the participant answer in the first column.

| **Participant answer** | C1 | C2 | C3 | C4 | C5 | C6 | C7 | C8 | C9 | C10 | C11 | C12 | C13 | C14 | C15 | C16 | C17 | C18 | C19 | C20 | C21 | C22 | C23 | C24 | C25 | C26 | C27 | C28 | C29 | C30 | C31 | C32 | C33 | C34 | C35 | C36 | C37 | C38 |
| --- | --- | --- | --- | --- | --- | --- | --- | --- | --- | --- | --- | --- | --- | --- | --- | --- | --- | --- | --- | --- | --- | --- | --- | --- | --- | --- | --- | --- | --- | --- | --- | --- | --- | --- | --- | --- | --- | --- |
| Repeated signs of poisoning | 1 |  |  |  |  |  |  |  |  |  |  |  |  |  |  |  |  |  |  |  |  |  |  |  |  |  |  |  |  |  |  |  |  |  |  |  |  | 1 |
| Vague complaints (so difficult to check), a long patient file, already visited multiple practices or referral central (also alternative medicine), large need for attention during or after consultation (calling/mailing often) |  | 1 | 1 |  |  |  |  |  |  |  |  |  |  |  |  |  |  |  |  |  |  |  |  |  |  |  |  |  |  |  |  |  |  |  |  |  |  |  |
| Repeating care demand, disproportionate seriousness |  |  | 1 | 1 |  |  |  |  |  |  |  |  |  |  |  |  |  |  |  |  |  |  |  |  |  |  |  |  |  |  |  |  |  |  |  |  |  |  |
| Lameness, itchiness, diarrhoea |  |  |  |  | 1 | 1 | 1 |  |  |  |  |  |  |  |  |  |  |  |  |  |  |  |  |  |  |  |  |  |  |  |  |  |  |  |  |  |  |  |
| Inexplicable phenomena that cannot be answered from additional examination, repeatedly phoning the clinic, specifically with the question to speak with the veterinarian, often visiting the practice |  | 1 | 1 |  |  |  |  | 1 |  |  |  |  |  |  |  |  |  |  |  |  |  |  |  |  |  |  |  |  |  |  |  |  |  |  |  |  |  |  |
| Repeated visits for small complaints, often visiting with a healthy animal, no serious trauma seen |  |  | 1 |  |  |  |  |  | 1 |  |  |  |  |  |  |  |  |  |  |  |  |  |  |  |  |  |  |  |  |  |  |  |  |  |  |  |  |  |
| Often visiting without a clear cause |  |  | 1 |  |  |  |  |  | 1 |  |  |  |  |  |  |  |  |  |  |  |  |  |  |  |  |  |  |  |  |  |  |  |  |  |  |  |  |  |
| Mentioning complaints in the animal that are not seen in physical examination |  | 1 |  |  |  |  |  |  |  |  |  |  |  |  |  |  |  |  |  |  |  |  |  |  |  |  |  |  |  |  |  |  |  |  |  |  |  |  |
| Multiple dogs (4-5) over 15 years with vague illnesses (allergy/immune-related complaints and pu-pd without cause), after which at a certain moment the request for euthanasia was made or the animal was rehomed/relinquished at a relatively young age |  | 1 |  |  |  | 1 |  |  |  | 1 | 1 | 1 | 1 |  |  |  |  |  |  |  |  |  |  |  |  |  |  |  |  |  |  |  |  |  |  |  |  |  |
| More than average consultation frequency; reporting clinical complaints that are not verifiable or relatable to each other ('diffused/difficult anamnesis'). Absence of abnormalities in physical or additional examination |  | 1 | 1 |  |  |  |  |  |  |  |  |  |  | 1 |  |  |  |  |  |  |  |  |  |  |  |  |  |  |  |  |  |  |  |  |  |  |  |  |
| Frequent visits to the clinic, repeatedly new or changing symptoms, symptoms mentioned as a disease is described |  |  | 1 |  |  |  |  |  |  |  |  |  |  |  | 1 | 1 |  |  |  |  |  |  |  |  |  |  |  |  |  |  |  |  |  |  |  |  |  |  |
| Complaints that are lacking at admission, very many/often complaints that remain inexplicable or are prevalent at very high frequency |  | 1 |  |  |  |  |  |  |  |  |  |  |  |  |  |  | 1 | 1 |  |  |  |  |  |  |  |  |  |  |  |  |  |  |  |  |  |  |  |  |
| Inexplicable wounds or fractures |  |  |  |  |  |  |  |  |  |  |  |  |  |  |  |  |  |  | 1 |  |  |  |  |  |  |  |  |  |  |  |  |  |  |  |  |  |  |  |
| If something else is the matter all the time and results from examination are inconsistent with the story |  | 1 |  |  |  |  |  |  |  |  |  |  |  |  | 1 |  |  |  |  |  |  |  |  |  |  |  |  |  |  |  |  |  |  |  |  |  |  |  |
| Poor healing process, incorrect use of medication |  |  |  |  |  |  |  |  |  |  |  |  |  |  |  |  |  |  |  | 1 | 1 |  |  |  |  |  |  |  |  |  |  |  |  |  |  |  |  |  |
| Frequent visits, anamnesis inconsistent with physical examination and repeatedly changing complaints |  | 1 | 1 |  |  |  |  |  |  |  |  |  |  |  | 1 |  |  |  |  |  |  |  |  |  |  |  |  |  |  |  |  |  |  |  |  |  |  |  |
| Clinical picture and anamnesis are inconsistent, treatment results are poor; highly concerned owner with odd questions |  | 1 |  |  |  |  |  |  |  |  |  |  |  |  |  |  |  |  |  | 1 |  | 1 |  |  |  |  |  |  |  |  |  |  |  |  |  |  |  |  |
| People that want to visit very often |  |  | 1 |  |  |  |  |  |  |  |  |  |  |  |  |  |  |  |  |  |  |  |  |  |  |  |  |  |  |  |  |  |  |  |  |  |  |  |
| Repeated visits with a clinical deviant picture and abnormal diagnostical examinations in a short time frame |  | 1 | 1 |  |  |  |  |  |  |  |  |  |  |  |  |  |  |  |  |  |  |  |  |  |  |  |  |  |  |  |  |  |  |  |  |  |  |  |
| Returning very often with an animal, an animal in which you can not find anything wrong and that comes across as healthy |  | 1 |  |  |  |  |  |  | 1 |  |  |  |  |  |  |  |  |  |  |  |  |  |  |  |  |  |  |  |  |  |  |  |  |  |  |  |  |  |
| Repeatedly returning owner with all kinds of suspicions of conditions, while nothing is wrong with the animal in question |  | 1 |  |  |  |  |  |  | 1 |  |  |  |  |  |  |  |  |  |  |  |  |  |  |  |  |  |  |  |  |  |  |  |  |  |  |  |  |  |
| Discussed complaints are not visible in clinical picture; too often suffering from a certain condition |  | 1 |  |  |  |  |  |  |  |  |  |  |  |  |  |  |  | 1 |  |  |  |  |  |  |  |  |  |  |  |  |  |  |  |  |  |  |  |  |
| Repeated visits and not being able to find anything in the animal |  |  |  |  |  |  |  |  | 1 |  |  |  |  |  |  |  |  |  |  |  |  |  |  |  |  |  |  |  |  |  |  |  |  |  |  |  |  |  |
| Highly frequent appointments, unlikely injury |  |  | 1 |  |  |  |  |  |  |  |  |  |  |  |  |  |  |  |  |  |  |  |  |  |  |  |  |  |  |  |  |  |  |  |  |  |  |  |
| Too often requesting visits; wounds; gastro-intestinal issues |  |  | 1 |  |  |  | 1 |  |  |  |  |  |  |  |  |  |  |  | 1 |  |  |  |  |  |  |  |  |  |  |  |  |  |  |  |  |  |  |  |
| Above average visiting rate, requests by owner for (invasive) examination that are inappropriate for the situation |  |  | 1 |  |  |  |  |  |  |  |  |  |  |  |  |  |  |  |  |  |  |  | 1 |  |  |  |  |  |  |  |  |  |  |  |  |  |  |  |
| Repeated visits for various illnesses |  |  | 1 |  |  |  |  |  |  |  |  |  |  |  | 1 |  |  |  |  |  |  |  |  |  |  |  |  |  |  |  |  |  |  |  |  |  |  |  |
| Repeated veterinary visits without demonstratable medical causes |  |  | 1 |  |  |  |  |  | 1 |  |  |  |  |  |  |  |  |  |  |  |  |  |  |  |  |  |  |  |  |  |  |  |  |  |  |  |  |  |
| Injuries that are insufficiently explicable, or for example famishing an animal (that it eats very well in the clinic for example) |  |  |  |  |  |  | 1 |  |  |  |  |  |  |  |  |  | 1 |  | 1 |  |  |  |  |  |  |  |  |  |  |  |  |  |  |  |  |  |  |  |
| Repeated complaints, not based on facts |  | 1 | 1 |  |  |  |  |  |  |  |  |  |  |  |  |  |  |  |  |  |  |  |  |  |  |  |  |  |  |  |  |  |  |  |  |  |  |  |
| Unnecessarily high medication administration, many veterinary visits, not taking advise from personnel |  |  | 1 |  |  |  |  |  |  |  |  |  |  |  |  |  |  |  |  |  | 1 |  |  | 1 |  |  |  |  |  |  |  |  |  |  |  |  |  |  |
| Weekly, near daily clinic visits with ill animals, often various complaints, both young and old animals; according to owner complaints, but not seen by professional; owner has diagnosed animal before the consultation |  | 1 | 1 |  |  |  |  |  |  |  | 1 |  |  |  | 1 |  |  | 1 |  |  |  |  |  |  | 1 |  |  |  |  |  |  |  |  |  |  |  |  |  |
| People visiting with animals that did better, but whose wounds are open suddenly; animals that were fine first, owners visit, later physical issues can be seen in dog/cat |  |  |  |  |  |  |  |  |  |  |  |  |  |  |  |  |  |  |  | 1 |  |  |  |  |  | 1 |  |  |  |  |  |  |  |  |  |  |  |  |
| Suspicious injuries and an owner that asks many questions/ is lengthy in conversation/ often contacts the clinic without visiting or showing interest in solutions for fast recovery of the animal |  |  |  |  |  |  |  |  |  |  |  |  |  |  |  |  |  |  | 1 |  |  | 1 |  | 1 |  |  |  |  |  |  |  |  |  |  |  |  |  |  |
| Owners that continuously call with all kinds of questions on a possible condition and often request medication by phone; or that always visit, but that the animal then is fine |  |  | 1 |  |  |  |  |  | 1 |  |  |  |  |  |  |  |  |  |  |  |  | 1 |  |  |  |  | 1 |  |  |  |  |  |  |  |  |  |  |  |
| Odd injuries, inexplicable and unrepeated symptoms |  | 1 |  |  |  |  |  |  |  |  |  |  |  |  |  |  |  |  | 1 |  |  |  |  |  |  |  |  |  |  |  |  |  |  |  |  |  |  |  |
| Many visits, overly concerned, insisting, close contact with personnel, very friendly but also very good at complaining |  |  | 1 |  |  |  |  |  |  |  |  |  |  |  |  |  |  |  |  |  |  | 1 |  |  |  |  |  | 1 | 1 |  |  |  |  |  |  |  |  |  |
| Healthy dog of which the owner continuously calls that something is the matter |  |  |  |  |  |  |  |  | 1 |  |  |  |  |  |  |  |  |  |  |  |  |  |  |  |  |  |  |  |  |  |  |  |  |  |  |  |  |  |
| If an animal repeatedly suffers from something different |  |  |  |  |  |  |  |  |  |  |  |  |  |  | 1 |  |  |  |  |  |  |  |  |  |  |  |  |  |  |  |  |  |  |  |  |  |  |  |
| Too emotionally involved, insisting on hospitalisation without ground, wanting to have the animal submitted too long, social media behaviour, often underfed or overfed animals; bringing forward internet-derived diagnoses of very rare illnesses of which all of a sudden the symptoms correspond; blaming the veterinarian if an animal is not recovering; visiting many different vets/specialists and no one can help their animal |  |  | 1 |  |  |  | 1 |  |  |  |  |  |  |  |  | 1 |  |  |  |  |  | 1 |  |  | 1 |  |  | 1 |  | 1 | 1 | 1 |  |  |  |  |  |  |
| Unclear story about the causes of fractures; suspicion of intoxication of animals that reside only at home; highly deviant behaviour of owners at discussion of the possible causes of a condition | 1 |  |  |  |  |  |  |  |  |  |  |  |  |  |  |  |  |  | 1 |  |  |  |  |  |  |  |  |  |  |  |  |  | 1 |  |  |  |  |  |
| Injury doesn't fit the story or anamnesis; omission of treatment by owner that results in unnecessary suffering of the animal |  |  |  |  |  |  |  |  |  |  |  |  |  |  |  |  |  |  | 1 |  |  |  |  | 1 |  |  |  |  |  |  |  |  |  |  |  |  |  |  |
| Repeated calls, self medicating (doctoring), ordering medication online by themselves, doubting diagnoses |  |  | 1 |  |  |  |  |  |  |  |  |  |  |  |  |  |  |  |  |  |  |  |  |  |  |  | 1 |  |  |  |  |  |  | 1 | 1 |  |  |  |
| Skin damage without other complaints, very frequent visits |  |  | 1 |  |  |  |  |  |  |  |  |  |  |  |  |  |  |  | 1 |  |  |  |  |  |  |  |  |  |  |  |  |  |  |  |  |  |  |  |
| No signs of fungal infection at clinical examination, no fur or skin damage (cat), but owner maintains that cat has fungus related issues |  | 1 |  |  |  |  |  |  |  |  |  |  |  |  |  |  |  |  |  |  |  |  |  |  |  |  |  |  |  |  |  |  |  |  |  |  |  |  |
| Unexplainable illness symptoms, of which none are findable in the patient, or constantly changing complaints |  | 1 |  |  |  |  |  |  |  |  |  |  |  |  |  |  |  |  |  |  |  |  |  |  |  |  |  |  |  |  |  |  |  |  |  |  |  |  |
| No symptoms, but an owner that sees a lot at home; or broken bones with an odd story |  | 1 |  |  |  |  |  |  |  |  |  |  |  |  |  |  |  |  | 1 |  |  |  |  |  |  |  |  |  |  |  |  |  |  |  |  |  |  |  |
| Repeated visits with changing complaints, while you cannot find anything during the animal's examination |  | 1 |  |  |  |  |  |  |  |  |  |  |  |  | 1 |  |  |  |  |  |  |  |  |  |  |  |  |  |  |  |  |  |  |  |  |  |  |  |
| Owner visits with an ill animal, animal is clinically healthy |  | 1 |  |  |  |  |  |  |  |  |  |  |  |  |  |  |  |  |  |  |  |  |  |  |  |  |  |  |  |  |  |  |  |  |  |  |  |  |
| Don't know, repeated lameness perhaps? |  |  |  |  | 1 |  |  |  |  |  |  |  |  |  |  |  |  |  |  |  |  |  |  |  |  |  |  |  |  |  |  |  |  |  |  |  |  |  |
| Continuously ill, without a clear cause; no complaints at the vet, but ill according to the owner; treating the animal before visiting and then complaining that one has too little medication to finish the treatment |  | 1 |  |  |  |  |  |  |  |  |  |  |  |  |  |  |  |  |  |  | 1 |  |  |  |  |  |  |  |  |  |  |  |  |  | 1 |  |  |  |
| Calling everyday on unnecessary points; wanting medication without the animal needing it |  |  | 1 |  |  |  |  |  |  |  |  |  |  |  |  |  |  |  |  |  |  |  |  |  |  |  | 1 |  |  |  |  |  |  |  |  |  |  |  |
| Negative tests, no clear diagnose; no improvement on medication |  | 1 |  |  |  |  |  |  |  |  |  |  |  | 1 |  |  |  |  |  |  | 1 |  |  |  |  |  |  |  |  |  |  |  |  |  |  |  |  |  |
| Successive clinical picture, with the former hardly solved or when clinical examination reveals no deviation |  | 1 |  |  |  |  |  |  |  |  |  |  |  | 1 | 1 |  |  |  |  |  |  |  |  |  |  |  |  |  |  |  |  |  |  |  |  |  |  |  |
| Owners with a personality disorder that at least once a year are in the clinic crying, next to a healthy animal |  |  |  |  |  |  |  |  | 1 |  |  |  |  |  |  |  |  |  |  |  |  |  |  |  |  |  |  |  |  |  |  |  |  |  |  |  | 1 |  |
| Incoherent story over the dog's ailment and all ailments of the person, family/relations, animals that are not patient in the clinic |  | 1 |  |  |  |  |  |  |  |  |  |  |  |  |  |  |  |  |  |  |  | 1 |  |  |  |  |  |  |  |  |  |  |  |  |  |  |  | 1 |
| Visiting often, calling often, while the animal is not clinically ill; requests for further examination |  |  | 1 |  |  |  |  |  | 1 |  |  |  |  |  |  |  |  |  |  |  |  |  | 1 |  |  |  |  |  |  |  |  |  |  |  |  |  |  |  |
| Animals with multiple ailments at once (unreasonably many, sometimes 4-5 diagnoses); owners that visit multiple clinics; owners that are not settling with simple conclusions based on a clinical picture; owners whom themselves work in a (health) care profession |  |  | 1 |  |  |  |  |  |  |  |  |  |  |  |  |  |  | 1 |  |  |  |  |  | 1 |  |  |  |  |  |  |  |  |  |  |  | 1 |  |  |
| Vague complaints, incoherent story |  | 1 |  |  |  |  |  |  |  |  |  |  |  |  |  |  |  |  |  |  |  |  |  |  |  |  |  |  |  |  |  |  |  |  |  |  |  |  |
| If the owners visit repeatedly for vague complaints |  | 1 | 1 |  |  |  |  |  |  |  |  |  |  |  |  |  |  |  |  |  |  |  |  |  |  |  |  |  |  |  |  |  |  |  |  |  |  |  |
| No complaints, clinically not ill, while the owner's story is different |  | 1 |  |  |  |  |  |  | 1 |  |  |  |  |  |  |  |  |  |  |  |  |  |  |  |  |  |  |  |  |  |  |  |  |  |  |  |  |  |
| If the owners visit often with vague complaints |  | 1 | 1 |  |  |  |  |  |  |  |  |  |  |  |  |  |  |  |  |  |  |  |  |  |  |  |  |  |  |  |  |  |  |  |  |  |  |  |
| Repeated unnecessary return visits |  |  | 1 |  |  |  |  |  |  |  |  |  |  |  |  |  |  |  |  |  |  |  |  |  |  |  |  |  |  |  |  |  |  |  |  |  |  |  |
| Anamnesis not fitting with the findings |  | 1 |  |  |  |  |  |  |  |  |  |  |  |  |  |  |  |  |  |  |  |  |  |  |  |  |  |  |  |  |  |  |  |  |  |  |  |  |
| Repeated symptoms that are not explained by the diagnosis |  | 1 |  |  |  |  |  |  |  |  |  |  |  |  |  |  |  |  |  |  |  |  |  |  |  |  |  |  |  |  |  |  |  |  |  |  |  |  |
| Continuing owner complaint on the health of the animal without observable clinical symptoms |  |  |  |  |  |  |  |  | 1 |  |  |  |  |  |  |  |  |  |  |  |  |  |  |  |  |  |  |  |  |  |  |  |  |  |  |  |  |  |
| Frequent visits with the same or different unlikely/rare symptoms in light of breed/age, etc. |  | 1 | 1 |  |  |  |  |  |  |  |  |  |  |  | 1 |  |  |  |  |  |  |  |  |  |  |  |  |  |  |  |  |  |  |  |  |  |  |  |
| Returning complaints, worsening despite treatment, multiple problems, owner-mentioned symptoms inconsistent with clinical examination/ diagnostics |  | 1 |  |  |  |  |  |  |  |  |  |  |  |  | 1 |  |  |  |  | 1 |  |  |  |  |  |  |  |  |  |  |  |  |  |  |  |  |  |  |
| Inexplicable trauma, unlikeliness of returning complaints |  |  |  |  |  |  |  |  |  |  |  |  |  |  | 1 |  |  |  | 1 |  |  |  |  |  |  |  |  |  |  |  |  |  |  |  |  |  |  |  |
| Frequent veterinary visits, ailments not provable, physical examination not aligned with owner's information, deviating general mental impression of owner |  | 1 | 1 |  |  |  |  |  |  |  |  |  |  | 1 |  |  |  |  |  |  |  |  |  |  |  |  |  |  |  |  |  |  |  |  |  |  | 1 |  |
| Owner complaints that are not seen in the animal, e.g. poor eating, no weight loss |  | 1 |  |  |  |  | 1 |  |  |  |  |  |  |  |  |  |  |  |  |  |  |  |  |  |  |  |  |  |  |  |  |  |  |  |  |  |  |  |
| Visiting very often, with vague complaints/anamnesis, different complaints at each visit, not fitting the animal/ situation, odd disease presentation, unlogic |  | 1 | 1 |  |  |  |  |  |  |  |  |  |  |  | 1 |  |  |  |  |  |  |  |  |  |  |  |  |  |  |  |  |  |  |  |  |  |  |  |

**Codes:**

| Code | Label | Category |
| --- | --- | --- |
| C1 | Signs of poisoning | Disease and condition characteristics |
| C2 | Vague or inexplicable complaints - difficult to check or confirm medically | Complaint characteristics |
| C3 | Recurrent contact moments/ visits with one or multiple professionals | Contact characteristics |
| C4 | Uncommon seriousness of complaints | Complaint characteristics |
| C5 | Signs of lameness | Disease and condition characteristics |
| C6 | Signs of itchiness, allergy, relating to the immune system | Disease and condition characteristics |
| C7 | Signs of diarrhoea/GI and/or starving - famished animal | Disease and condition characteristics |
| C8 | Asking specifically to talk to the veterinarian | Contact characteristics |
| C9 | Repetitive small complaints/visits with a healthy animal | Complaint characteristics |
| C10 | Sign PU-PD (Polyuria-polydipsia) | Disease and condition characteristics |
| C11 | Multiple animals | Animal keeping characteristics |
| C12 | Request for euthanasia at relatively young age | Animal keeping characteristics |
| C13 | Rehoming/relinquishment of animals | Animal keeping characteristics |
| C14 | Clinical abnormality absence upon medical/laboratory/additional checks | Complaint characteristics |
| C15 | (Unlikely) repeatedly new or changing symptoms, multiple problems | Complaint characteristics |
| C16 | Mentioning symptoms in the manner that a disease is described | Complaint characteristics |
| C17 | Complaints that disappear upon clinical admission | Complaint characteristics |
| C18 | High frequency of complaints | Complaint characteristics |
| C 19 | Inexplicable injuries/uncommon fractures | Disease and condition characteristics |
| C20 | Slowed healing/recovery or even worsening despite treatment | Treatment course characteristics |
| C21 | Incorrect administration of medication/ no improvement on medication | Treatment course characteristics |
| C22 | High concern ventilation by owner and/ or lengthy in conversations | Contact characteristics |
| C23 | Requests for unnecessary (invasive) examination | Contact characteristics |
| C24 | Unwilling to take advice from a veterinarian/ no interest in solutions that will aid quick recovery | Contact characteristics |
| C25 | Owner diagnosed animal before appointment | Complaint characteristics |
| C26 | Animal is reportedly ill, when owners visit no clinical findings at first, but clinical findings present at a second visit | Treatment course characteristics |
| C27 | Asking for medication by phone, insisting on medication prescription, or ordering it via internet by themselves | Contact characteristics |
| C28 | Insisting, being friendly but also complaining | Contact characteristics |
| C29 | Being in close contact with personnel | Contact characteristics |
| C30 | Insisting on hospitalisation or longer hospital admission | Contact characteristics |
| C31 | Social media behaviour | Contact characteristics |
| C32 | Blaming the veterinarian if the animal does not recover and/ or claiming that no one can help the animal | Contact characteristics |
| C33 | Deviant behaviour of owner(s) upon discussion of possible causes of a condition | Contact characteristics |
| C34 | Challenging the diagnosis | Contact characteristics |
| C35 | Self-medicating (doctoring) the animal | Treatment course characteristics |
| C36 | A profession in (health)care of client | Owner characteristics |
| C37 | Client with personality disorder (characteristics)/Deviance in mental health state | Owner characteristics |
| C38 | Conversations by client on medical issues of others than animal e.g. relatives, self, other animals than patient | Contact characteristics |
