# Supplementary material for: Animal abuse by falsification–Recognition amongst the veterinary profession in The Netherlands
Source: PLoS One. 2026 Apr 8;21(4):e0345067. doi: 10.1371/journal.pone.0345067 (PMC13061241; doi:10.1371/journal.pone.0345067)
Supplement: S5 Table — (DOCX) [file pone.0345067.s005.docx]

**S5 Table. What would aid in reporting Animal Abuse by Falsification (AAF) as mentioned by participants in the open question on reporting: ‘What would help you to report AAF?’**

The C’s (for ‘code’) with a number in the top row correspond with the codes assigned and listed in the table at the bottom of this file. When a ‘1’ is visible in a field, this indicates that the code was attributed based on the participant answer in the first column.

| **Participant answer** | **C1** | **C2** | **C3** | **C4** | **C5** | **C6** | **C7** | **C8** | **C9** | **C10** | **C11** | **C12** | **C13** | **C14** | **C15** | **C16** | **C17** | **C18** | **C19** | **C20** | **C21** | **C22** |
| --- | --- | --- | --- | --- | --- | --- | --- | --- | --- | --- | --- | --- | --- | --- | --- | --- | --- | --- | --- | --- | --- | --- |
| More information over the phenomenon | 1 |  |  |  |  |  |  |  |  |  |  |  |  |  |  |  |  |  |  |  |  |  |
| More information over the condition | 1 |  |  |  |  |  |  |  |  |  |  |  |  |  |  |  |  |  |  |  |  |  |
| Easy access, low time investment, no need to discuss with owner yourself |  | 1 | 1 | 1 |  |  |  |  |  |  |  |  |  |  |  |  |  |  |  |  |  |  |
| Guidelines for 'red flags' |  |  |  |  | 1 |  |  |  |  |  |  |  |  |  |  |  |  |  |  |  |  |  |
| Knowing whom to actually contact: police? neighbourhood police? |  |  |  |  |  | 1 |  |  |  |  |  |  |  |  |  |  |  |  |  |  |  |  |
| Ability to recognise with certainty, no false accusation |  |  |  |  |  |  | 1 |  |  |  |  |  |  |  |  |  |  |  |  |  |  |  |
| To know what such a process looks like |  |  |  |  |  | 1 |  |  |  |  |  |  |  |  |  |  |  |  |  |  |  |  |
| More knowledge/certainty of the topic and how to approach/identify the owner |  |  |  |  |  |  | 1 | 1 |  |  |  |  |  |  |  |  |  |  |  |  |  |  |
| Beter understanding of the psychological condition itself and guidelines for recognition |  |  |  |  |  |  |  | 1 |  |  |  |  |  |  |  |  |  |  |  |  |  |  |
| More information on the signs |  |  |  |  |  |  |  |  | 1 |  |  |  |  |  |  |  |  |  |  |  |  |  |
| Flow chart to achieve a near certain diagnosis of animal abuse by illness falsification in cooperation with a psychologist/physician |  |  |  |  |  |  |  |  |  | 1 |  |  |  |  |  |  |  |  |  |  |  | 1 |
| Reporting centre |  | 1 |  |  |  |  |  |  |  |  |  |  |  |  |  |  |  |  |  |  |  |  |
| Where to report |  |  |  |  |  |  |  |  |  |  | 1 |  |  |  |  |  |  |  |  |  |  |  |
| Examples of what people do to animals |  |  |  |  |  |  |  |  |  |  |  | 1 |  |  |  |  |  |  |  |  |  |  |
| Protocol |  |  |  |  | 1 |  |  |  |  |  |  |  |  |  |  |  |  |  |  |  |  |  |
| Knowing that action will be taken |  |  |  |  |  |  |  |  |  |  |  |  | 1 |  |  |  |  |  |  |  |  |  |
| More information on what to attend to |  |  |  |  |  |  |  | 1 |  |  |  |  |  |  |  |  |  |  |  |  |  |  |
| More information | 1 |  |  |  |  |  |  |  |  |  |  |  |  |  |  |  |  |  |  |  |  |  |
| More knowledge of which path to follow |  |  |  |  | 1 | 1 |  |  |  |  |  |  |  |  |  |  |  |  |  |  |  |  |
| Better definition |  |  |  |  |  |  |  |  |  |  |  |  |  | 1 |  |  |  |  |  |  |  |  |
| Support from colleagues |  |  |  |  |  |  |  |  |  |  |  |  |  |  | 1 |  |  |  |  |  |  |  |
| A clear diagnosis |  |  |  |  |  |  |  |  |  |  |  |  |  |  |  | 1 |  |  |  |  |  |  |
| Experience, symptom delineation, familiarity |  |  |  |  |  |  |  | 1 |  |  |  |  |  |  |  |  | 1 |  |  |  |  |  |
| Knowing how and where, and what consequences are |  |  |  |  | 1 | 1 |  |  |  |  |  |  |  |  |  |  |  | 1 |  |  |  |  |
| More knowledge on recognition |  |  |  |  |  |  |  | 1 |  |  |  |  |  |  |  |  |  |  |  |  |  |  |
| A disclosure centre |  | 1 |  |  |  |  |  |  |  |  |  |  |  |  |  |  |  |  |  |  |  |  |
| More information | 1 |  |  |  |  |  |  |  |  |  |  |  |  |  |  |  |  |  |  |  |  |  |
| Help with recognition, you do not want to accuse someone wrongfully |  |  |  |  |  |  |  |  |  |  |  |  |  |  |  |  |  |  | 1 |  |  |  |
| Clear guidelines |  |  |  |  | 1 |  |  |  |  |  |  |  |  |  |  |  |  |  |  |  |  |  |
| It is difficult to ascertain; we can only have a suspicion; we are not equipped for psychological assessment |  |  |  |  |  |  | 1 |  |  |  |  |  |  |  |  |  |  |  |  |  |  |  |
| If my employer would agree |  |  |  |  |  |  |  |  |  |  |  |  |  |  | 1 |  |  |  |  |  |  |  |
| More knowledge, guidelines for signs and easy access of a reporting centre |  | 1 |  |  |  |  |  | 1 |  |  |  |  |  |  |  |  | 1 |  |  |  |  |  |
| More information/help line, more information on prevalence of the condition | 1 | 1 |  |  |  |  |  |  |  |  |  |  |  |  |  |  |  |  |  |  |  |  |
| Disclosure centre |  | 1 |  |  |  |  |  |  |  |  |  |  |  |  |  |  |  |  |  |  |  |  |
| A clearer image of when animal abuse through illness falsification applies |  |  |  |  |  |  |  | 1 |  |  |  |  |  |  |  |  |  |  |  |  |  |  |
| Insight into the signs of animal abuse through illness falsification |  |  |  |  |  |  |  | 1 |  |  |  |  |  |  |  |  |  |  |  |  |  |  |
| Identification characteristics at hand, more knowledge, it is at times vague, cannot say that a client is dishonest |  |  |  |  |  |  |  | 1 |  |  |  |  |  | 1 |  |  |  |  |  |  |  |  |
| Clarity on role in this and what consequences are for someone |  |  |  |  |  |  |  |  |  |  |  |  |  |  |  |  |  |  |  | 1 |  |  |
| More knowledge/guidance | 1 |  |  |  | 1 |  |  |  |  |  |  |  |  |  |  |  |  |  |  |  | 1 |  |
| More guidelines and a list of signs of animal abuse through illness falsification |  |  |  |  | 1 |  |  | 1 |  | 1 |  |  |  |  |  |  |  |  |  |  |  |  |
| Good contact with inspection bodies; that these are easily accessible, that is very good in our region, letting clinics know what to do | 1 | 1 |  |  | 1 | 1 |  |  |  |  |  |  |  |  |  |  |  |  |  |  |  |  |
| Online/telephonic reporting centre |  | 1 |  |  |  |  |  |  |  |  |  |  |  |  |  |  |  |  |  |  |  |  |
| Certainty of the case |  |  |  |  |  |  | 1 |  |  |  |  |  |  |  |  |  |  |  |  |  |  |  |
| Certainty that it regards illness falsification |  |  |  |  |  |  | 1 |  |  |  |  |  |  |  |  |  |  |  |  |  |  |  |
| Disclosure centre for consultation, more knowledge |  | 1 |  |  |  |  |  | 1 |  |  |  |  |  |  |  |  |  |  |  |  |  |  |
| More information on recognition |  |  |  |  |  |  |  | 1 |  |  |  |  |  |  |  |  |  |  |  |  |  |  |
| Knowing where I can go |  |  |  |  |  | 1 |  |  |  |  |  |  |  |  |  |  |  |  |  |  |  |  |
| Knowing where to report | 1 | 1 |  |  |  |  |  |  |  |  |  |  |  |  |  |  |  |  |  |  |  |  |
| Governing body less irritating behaviour |  |  |  |  |  |  |  |  |  |  |  |  | 1 |  |  |  |  |  |  |  |  |  |
| That there is a clear guideline to recognise it |  |  |  |  | 1 |  |  | 1 |  |  |  |  |  |  |  |  |  |  |  |  |  |  |
| If known which physician or mental health doctor can be phoned and if good and fitting care is available for people with mental health issues |  |  |  |  |  |  |  |  |  |  |  |  |  |  |  |  |  |  |  |  |  | 1 |
| Contact with other veterinarians or physicians that were in contact with the client |  |  |  |  |  |  |  |  |  |  |  |  |  |  | 1 |  |  |  |  |  |  | 1 |
| Consultation with the client's physician |  |  |  |  |  |  |  |  |  |  |  |  |  |  |  |  |  |  |  |  |  | 1 |
| More awareness: with children the trajectory seems clearer, with animals it is often a little strange to make such assumptions, as it is costly to visit the vet each week; clear checklist to document suspicions and to report - then more cases of animal abuse by illness falsification would surface |  | 1 |  |  |  |  |  | 1 |  | 1 |  |  |  |  |  |  |  |  |  |  |  | 1 |
| More information known | 1 |  |  |  |  |  |  |  |  |  |  |  |  |  |  |  |  |  |  |  |  |  |
| More awareness |  |  |  |  |  |  |  | 1 |  |  |  |  |  |  |  |  |  |  |  |  |  |  |
| A flowchart/decision taking instrument |  |  |  |  |  |  |  |  |  | 1 |  |  |  |  |  |  |  |  |  |  |  |  |
| Step-by-step plan |  |  |  |  | 1 |  |  |  |  |  |  |  |  |  |  |  |  |  |  |  |  |  |
| A central point of contact and funding to confirm a possible suspicion via additional examination |  | 1 | 1 |  |  |  |  |  |  |  |  |  |  |  |  |  |  |  |  |  |  |  |
| More knowledge on both the condition as on the possibilities of reporting it |  |  |  |  |  |  |  | 1 |  |  | 1 |  |  |  |  |  |  |  |  |  |  |  |
| Knowing where to report a suspicion |  |  |  |  |  |  |  |  |  |  | 1 |  |  |  |  |  |  |  |  |  |  |  |
| Better explanation on which organisation to contact |  |  |  |  |  |  |  |  |  |  | 1 |  |  |  |  |  |  |  |  |  |  |  |
| More information on where to report and when it regards AAF, you do not want to report just like that in case of suspicion |  |  |  |  |  |  | 1 | 1 |  |  | 1 |  |  |  |  |  |  |  |  |  |  |  |

**Codes:**

| **Code** | **Label** | **Category** |
| --- | --- | --- |
| C1 | More information | Information |
| C2 | (Easy access to) reporting centre/disclosure office | Guidelines and protocol |
| C3 | Low time investment/ financial support for additional examination | Support |
| C4 | No need to discuss with client yourself | Support |
| C5 | Guidelines/ protocol | Guidelines and protocol |
| C6 | Knowing whom to contact for help/ knowing how the process of reporting works | Information |
| C7 | Certainty of recognition/ no trust breach/ no false accusation risk | Assurance |
| C8 | More knowledge on topic and owner identification | Information |
| C9 | More knowledge on signs | Information |
| C10 | Flow chart for near certain diagnosis/ guide for documentation of signs and symptoms/ list of signs of the condition | Clarity on condition (recognition) |
| C11 | (Knowing) where to report | Guidelines and protocol |
| C12 | Examples of what people with the condition do to animals | Clarity on condition (recognition) |
| C13 | Knowing that action is taken upon reporting | Support |
| C14 | Better definition | Clarity on condition (recognition) |
| C15 | Support from colleagues/ employer | Support |
| C16 | A clear diagnosis (opportunity) | Clarity on condition (recognition) |
| C17 | Experience and familiarity with the condition | Information |
| C18 | Knowledge on consequences | Information |
| C19 | Assistance on recognition as to not incorrectly accuse a person | Support |
| C20 | Clarity on roles in the process and consequences for the involved | Guidelines and protocol |
| C21 | More support | Support |
| C22 | Opportunity to consult psychologist/ physician of client | Support |
