# Supplementary material for: Animal abuse by falsification–Recognition amongst the veterinary profession in The Netherlands
Source: PLoS One. 2026 Apr 8;21(4):e0345067. doi: 10.1371/journal.pone.0345067 (PMC13061241; doi:10.1371/journal.pone.0345067)
Supplement: Attachment — Submitted filename: Response to Reviewers.docx [file pone.0345067.s008.docx]

Utrecht, February 6^th^, 2026

Dear Academic Editors,

Thank you to you and the reviewers for reviewing our manuscript titled ‘Animal abuse by falsification – recognition amongst the veterinary profession in The Netherlands’. Below we address each point raised.

Please address all correspondence concerning this manuscript to me at i.r.vanherwijnen@uu.nl. Thank you for your consideration of this manuscript for a possible publication in PLoS ONE.

Sincerely,

Ineke R. van Herwijnen Ph.D.

i.r.vanherwijnen@uu.nl

Division of Animals in Science and Society, Faculty of Veterinary Medicine, Department of Population Health Sciences, Utrecht University, Utrecht, The Netherlands

**Response to each point raised:**

**2. Please include a new copy of Table S4 in your manuscript; the current table is difficult to read. Please follow the link for more information:** [**https://journals.plos.org/plosone/s/tables**](https://journals.plos.org/plosone/s/tables)

Thank you for suggesting this change, we have tried several variants. We have now added a supplement table with larger font size, while still keeping the data in one table. Keeping the information in one table was deemed beneficial by our test readers.

**Reviewer #1: The context of the article titled "Animal abuse by falsification – recognition amongst the veterinary profession in The Netherland" is highly appreciable in this era of the sociological need to assess human-animal interactions. The novelty is awesome. However, there is need to look at the following areas for possible amendments to add value to the write up:**

Thank you for this appreciated feedback, your reviewing time and suggestions, that helped us improve the manuscript.

**1. Line 33-35: There is need for modification and or recapping of the statement. The present forms is more of history than science!. What is the problem statement?. Highlight the gap in knowledge with this sentence.**
We have adapted our presentation of the topic at hand in the first three lines of the abstract.

**2. Line 38: What is vet tech? Please, write fully for readers who are not from your field to understand.**
Thank you for this suggestion, we have replaced ‘tech’ with ‘technician’ throughout.

**3.Line 50-51: What is the essence of the last sentence?**
We have removed the last sentence of the abstract.

**4. Line 78: .......is unknown. Where? Mention the name of the country**
We have added the USA region.

**5. Line 119: Participants**
We have adapted the title of this section.

**6. Line 123: What is vet tech??**
Thank you for this suggestion, we have replaced ‘tech’ with ‘technician’ throughout.

**7. The conclusion is not shown as a distinct subheadings like Results and Discussion. Please, carve the Conclusion out of the discussion.**
We have added a conclusion section.

**8. No DOI for the articles listed under the references.**

We have added the DOI to the articles in the reference list.

**Reviewer #2:**

Thank you for your time and comments that helped to improve our manuscript.

**Line 36-" perplexing presentation has been mentioned'' include reference so we know who is being referred to.**
Thank you for your suggestion. We are not customary to adding references to an abstract, but have provided references in the Introduction section.

**Line 46- Reporting of AAF in this sample was low. this statement is too short and should be merged with previous statement**
We have merged this sentence and the sentence thereafter.

**Line 47- does the 92% reported here inclusive of the participants that indicated they are unsure and have not seen such cases? I dont think these categories should be in this**
Reporting was indeed reportedly never done by 92.1% (N=81), 4.5% (N=4) reported AAF as animal abuse, 3.4% (N=3) didn’t know.

**line 59- recast the sentence like outcome may include instead of outcome may regard**
Thank you for this suggestion, which we adhered to.

**Line 67: among not amongst**
As far as we are aware, amongst is more commonly used in British English, but we have adapted to among, hoping this is in accord with PLOS One.

**line 74- cant start a sentence with or, please recast or merge**
Thank you for this suggestion, which we adhered to.

**line 77-82: recast the sentence to have a better flow,**
We have rewritten this section. It now reads: ‘A USA-based study indicated that the true incidence or prevalence of AAF is unknown [2]. The few available scientific studies on the topic, often present cases and do not provide epidemiological data [2]. An example is a UK-based study including six cases identified by veterinarians and three cases included by the researchers as possible AAF cases [13]. In this total of nine cases, signs were noted in the animals, that corresponded with signs in child abuse cases. The respective signs regarded the animal owner’s attention seeking behaviour, recovery of the animal upon owner-separation, serial incidents, real and likely factitious clinical signs, abnormal biochemical profiles, deliberately inflicted injury, tempering with surgical sites and switching from veterinary clinics [13].’

**Line 85- switching among**
This sentence was adapted.

**Line 178- majority were veterinarians delete was a**
This sentence was adapted.

**Lines 110 and 120- Repeating the same information. Can you please recast the sentence for better clarity to be like we recruited...................... via e mail and platform**
Unfortunately PLOS One requires the ethical statement to be formulated as is and our reading panel judged it necessary to present the information that you point at also at the second location to facilitate the readers.

**Line 164- reframe the sentence ''how likely they thought a case to regard AAF,'**
This sentence was adapted.

**line 200- 31.8% cannot be termed more since 51.2 % already indicated, please remove more and recast the sentence**
Thank you for pointing out the unclarity in our writing. We have rewritten this text as: ‘51.2% (N=45) indicated to likely see cases. 4.6% (N=4) indicated to certainly see cases, 12.5% (N=11) indicated to certainly not see cases in their clinic. A relatively high percentage of 31.8% (N=28) indicated ‘don’t know’.’

**-- Create a footnote for table 1, what is PU-PD**
Thank you for this suggestion. We added ‘polyuria-polydipsia’ between brackets behind the abbreviation.

**290-291- e.g. (4) what is the meaning of this? please clarify**
We have rewritten this text section, taking away the unclarity.

**Line 291- This, as some participants responded that they recognise as an indication of AAF'' please recast for clarity**
We have rewritten this text section, taking away the unclarity.

**Line 297-298-- statement is disjointed, please recast**
We have rewritten this text section, taking away the unclarity.

**line 304- please recast**

This text was adapted.
